# Revolutionizing Personalized Medicine: Synergy with Multi-Omics Data Generation, Main Hurdles, and Future Perspectives

**DOI:** 10.3390/biomedicines12122750

**Published:** 2024-11-30

**Authors:** Getnet Molla, Molalegne Bitew

**Affiliations:** 1 College of Veterinary Medicine, Jigjiga University, Jigjiga P.O. Box 1020, Ethiopia; 2 Bio and Emerging Technology Institute (BETin), Addis Ababa P.O. Box 5954, Ethiopia; molalegne23@yahoo.com

**Keywords:** precision medicine, genomics, transcriptomics, proteomics, metabolomics, epigenomics

## Abstract

The field of personalized medicine is undergoing a transformative shift through the integration of multi-omics data, which mainly encompasses genomics, transcriptomics, proteomics, and metabolomics. This synergy allows for a comprehensive understanding of individual health by analyzing genetic, molecular, and biochemical profiles. The generation and integration of multi-omics data enable more precise and tailored therapeutic strategies, improving the efficacy of treatments and reducing adverse effects. However, several challenges hinder the full realization of personalized medicine. Key hurdles include the complexity of data integration across different omics layers, the need for advanced computational tools, and the high cost of comprehensive data generation. Additionally, issues related to data privacy, standardization, and the need for robust validation in diverse populations remain significant obstacles. Looking ahead, the future of personalized medicine promises advancements in technology and methodologies that will address these challenges. Emerging innovations in data analytics, machine learning, and high-throughput sequencing are expected to enhance the integration of multi-omics data, making personalized medicine more accessible and effective. Collaborative efforts among researchers, clinicians, and industry stakeholders are crucial to overcoming these hurdles and fully harnessing the potential of multi-omics for individualized healthcare.

## 1. Introduction

A medical concept termed “personalized medicine” (PM), often referred to as precision medicine (PM), divides patients into various groups according to their expected response or risk of contracting a disease. Thus, personalized medicine represents a transformative strategy in the medical field, focusing on tailoring medical treatment to the individual characteristics of each patient. This mechanism customizes medical choices, procedures, interventions, and/or goods to the level of each patient individually [1]. The idea of personalized medicine primarily works by considering individual variability in genomic content, environment, and lifestyle status; it aims to provide the right treatment to the right patient at the right time [2,3].

Both concepts aim to optimize treatment by moving away from the traditional “one-size-fits-all” approach in medicine. Precision medicine is often considered a subset of personalized medicine, as it uses genetic and molecular information to develop more targeted therapies, focusing on identifying subgroups of patients with specific characteristics (such as genetic mutations) that make them more likely to respond to certain treatments. Personalized medicine, on the other hand, takes a broader approach, incorporating multiple factors beyond biology to tailor treatment to individual patients [4].

The explosive growth in our knowledge of genomics, transcriptomics, proteomics, metabolomics and the molecular origins of disease is making its way to doctors’ offices, patient bedsides, and medicine cabinets of ordinary people [5]. This way, physicians can guide treatments by using genomic, mRNA, protein, and metabolic markers in a way that they never have before, leading to more improved, effective, and individualized patient care. Since mapping the human genome in 2003, the pace of discovery, product development, and clinical adoption of what we have come to know as “personalized medicine” has accelerated [6,7]. It has been ten years since the term ‘personalized medicine’ was first used in the context that we understand today, and the intervening years have seen a dramatic expansion and progression in its prevalence in the scientific community, medias, and a broad recognition in the wider population [8]. These advancements allow a more comprehensive understanding of individual patient profiles, resulting in more targeted and effective therapies.

As stated by the European Parliamentary Research Service (EPRS 2015), it refers to an “emerging—evolving approach to medicine” that applies scientific insights into the genetic and molecular basis of health and disease made possible by the sequencing of the human genome to guide decisions in regard to the prediction, prevention, diagnosis, and treatment of disease [9,10]. Similarly, the National Cancer Institute (NIH) underlined the three main purposes of personalized medicine: to prevent, to diagnose and treat disease by using specific information about each patient’s genes, proteins, and environment [10,11].

The human genome essentially forms the foundation of personalized medicine, regarded as the next generation of diagnosis and treatment [12]. Cutting-edge biochemical advances such as single-nucleotide polymorphisms (SNPs), genotyping, and biochips have made personalized medicine a reality, justifying the use of the terminology in the last few decades. Variations such as SNPs, insertions and deletions, structural variants, and copy number variations in the human genome play a significant role in the manifestation and progression of diseases like cancer, diabetes, neurodegenerative, and cardiovascular diseases [13,14].

Additionally, the President’s Council of Advisors on Science and Technology Executive Office of the President of the United States, with a report on September 2008, has clarified that the term personalized medicine “does not entail the creation of drugs or medical devices that are uniquely tailored to each patient, but rather the ability to categorize individuals into subpopulations based on differences in their susceptibility to a particular disease or their response to specific treatments. Preventive or therapeutic interventions can then be concentrated on those who will benefit, sparing expense and side effects for those who will not” [10,15].

By linking together diverse datasets to uncover previously unknown causal pathways and correlations, big data enables far greater precision and customization than was ever achievable before [16]. Recent scientific advancements in high-throughput, high-resolution data-generating technologies allow cost-effective analysis of big datasets on individual health. However, analyzing and integrating such vast amounts of information requires new computational approaches, including faster and more integrated processors, larger computer memories, improved sensors, sophisticated algorithms, methodologies, and cloud computing. These advancements have the potential to guide future clinical practice by delivering clinically useful information [13,17].

The development of big data approaches has enhanced the ability to investigate which aspects of biology exhibit functional and dysfunctional activity. However, for most clinical challenges, precision strategies remain aspirational. The difficulty lies in breaking down biology into its component parts, identifying which parts are measurable and relevant for selecting the best intervention, determining the patient population that will benefit, and pinpointing the optimal timing for intervention. This challenge cannot be overstated. However, the increasing use of hypothesis-free, big data approaches holds promise in helping us achieve this aspirational goal [18]. 

Theoretically, treatments’ focused on a person’s genetic profile should be more effective and have fewer adverse effects. But in practice, personalized medicine is often erratic and expensive, and often there are simpler solutions. Its practical implementation usually encounters significant challenges. In order to improve all of these obstacles, it also requires that people trust governments and companies with their genomic data while the regulatory environment around medicines remains ill-equipped to cope with therapies that are designed for just one individual person. Obtaining the safety and efficacy data necessary for regulatory approval typically entails conducting clinical trials involving hundreds, if not thousands, of participants. However, researchers are still trying, and it now seems there may be some genuine progress from time to time [19,20].

In the future, personalized medicine is likely to reduce drug-development costs, treatment expenses, and time, which will lead to improved and effective healthcare outcomes [21]. For personalized medicine to truly revolutionize the healthcare system, everyone from patients to regulatory authorities, researchers, and leaders must play a pivotal role by fostering new and innovative ideas, positive perspectives towards new technology, experimental diagnostics, and personalized care protocols [22]. All these efforts come from concern that the aforementioned bodies will transform personalized medicine into the next level of application in the healthcare system.

Nowadays, pharmaceutical industry leaders worldwide view precision medicine as a significant opportunity. Despite facing internal and external hurdles, only a few companies have successfully capitalized on its potential. Despite the promise of pharmacogenomics technologies to drive fundamental advances in biological sciences, such as discovering disease-causing genes and new therapeutic targets, industry concerns can arise regarding products designed with pharmacogenomics guidance for specific patient populations [23,24].

This concept paper explores the integration of pharmacogenomic testing and multi-omics data into personalized medicine as a new standard in the medical field. It highlights the potential benefits, challenges, and strategies for implementation, emphasizing the role of personalized medicine in enhancing medication quality control and therapeutic efficacy. Through literature reviews, case studies, and best practices, the paper provides insights into how pharmacogenomics testing could transform healthcare delivery in the future.

## 2. Historical Roots for the Development of Personalized Medicine

The history of personalized medicine is punctuated by significant milestones in genetics, technology, and clinical applications, shifting healthcare from a one-size-fits-all approach to a more individualized understanding of the molecular basis of health and disease and effective treatment strategies [25]. The concept of personalized medicine traces back to Hippocrates, who proposed that individual patient differences could impact treatment outcomes. However, its scientific foundations were established in the 20th century with the discoveries of DNA and the structure of the genetic code [26]. The usage of the term has risen in recent years thanks to the development of new diagnostic and informatics approaches that provide an understanding of the molecular basis of disease, comparative genomics. Furthermore, the Human Genome Project (HGP), com-pleted in 2003, was a monumental milestone that mapped the entire human genome, of-fering a reference for understanding genetic variations among individuals [27,28].

Over the last six decades, many studies have suggested that a significant portion of drug response variability is determined by genetics, along with diet, health condition, exposure to the environment, and a combined treatment regimen [29]. Advancements in understanding the molecular factors that underlie the impact of individual genetic constitution on disease and therapeutics have been significantly aided by developments in pharmacogenomics and pharmacogenetics [3]. Pharmacogenomics focuses on the genetic causes behind differences in how individuals respond to drugs, while pharmacogenetics studies how multiple variations within the genome affect responses to drug treatments [30].

In this case, drug treatment must be tailored to individual needs with a relatively predictable outcome in a range of geographically and ethnically diverse communities. From the early 1950s onwards, these findings of highly variable drug reactions led to a new scientific discipline called pharmacogenomics, integrating the fields of genetics, biochemistry, and pharmacology [31].

The development of pharmacogenomics stemmed from molecular medicine advances, seeking to comprehend the molecular mechanisms behind drug responses. Thus, personalized medicine is the name given to this application of research. Personalized medicine has yet to be fully understood by the average patient, but it is poised to eventually impact the entire healthcare system [32,33].

Today, personalized medicine has transformed cancer treatment. Progress in genomic sequencing and molecular diagnostics helps oncologists classify cancers with greater accuracy. This enables for more accurate diagnosis, prognosis, and anticipation of treatment response [34]. Targeted therapies, like *HER2* inhibitors for breast cancer and *EGFR* inhibitors for lung cancer, are created based on particular genetic mutations in cancers [35]. Technological advancements, such as next-generation sequencing (NGS) and bioinformatics, have sped up the analysis of genetic data and the identification of biomarkers for various disease conditions [36].

Personalized medicine is now being increasingly incorporated into daily clinical practice, especially in fields like oncology, cardiology, neurodegenerative diseases, and infectious diseases. This integration allows for more precise diagnosis, prognosis, and treatment strategies [37,38]. For example, one of the first successful targeted therapies was the anticancer drug imatinib. It is specifically designed for patients with chronic myelogenous leukemia (CML) who have an enzyme called *BCR-ABL* tyrosine kinase, produced by a cytogenetic abnormality known as the Philadelphia chromosome. Imatinib works by blocking the proliferation of CML cells with the mutated kinase, effectively reversing the cancerous effects of this abnormality [39]. 

Overall, the rise of personalized medicine has also been supported by advancements in health information technology, including the electronic processing and storage of patient data. The clinical adoption of personalized medicine, particularly through translational and clinical research, has been significantly influenced by these developments. Specifically, the implementation of electronic health records (EHRs), which store data on patient history, medications, test results, and demographics, has been crucial for integrating data from genetics and genomics research into clinical settings [40].

## 3. Personalized Medicine Transforms Healthcare

Throughout history, the medical profession has primarily been reactive, typically addressing illnesses and health crises as they occur rather than focusing on prevention. Often, treatments and cures are pursued only after a disease has already appeared. As a result, our efforts to treat major chronic diseases such as cancer, Alzheimer’s, and diabetes are often ineffective, inaccurate, or inconsistent due to our incomplete understanding of the molecular background and environmental factors that contribute to these conditions [41,42]. Due to this, the capacity to offer precision medicine to patients in routine clinical settings largely relies on the availability of molecular profiling tests [43].

Advances in genomics and biotechnology in the 21st century are enabling more personalized approaches to medicine, predicting disease risks, and tailoring treatments to individual genetic profiles. Today, there is increasing recognition of the significance of lifestyle factors in health, resulting in a greater emphasis on diet, exercise, and mental health as essential components of preventive medicine [44].

The concept of personalized medicine extends to innovative and transformative approaches in healthcare. Personalized healthcare leverages systems biology dynamics and predictive techniques to assess health risks and develop customized health strategies. These personalized approaches aim to help patients mitigate risks, prevent diseases, and treat them precisely if they occur. Today, the concepts of personalized healthcare are gaining widespread acceptance, with the Veterans Administration dedicating itself to personalized, proactive, patient-driven care for all veterans [45,46]. In certain cases, personalized healthcare can be tailored to the genetic makeup of the disease-causing agent rather than the patient’s genetic profile. Examples include drug-resistant bacteria or viruses [47].

Personalized medicine, which is based on a patient’s unique genetic profile, is starting to overcome the limitations of conventional medicine. Conventional medicine often employs a one-size-fits-all approach, designing treatments for the average patient, which can result in varying effectiveness and treatment outcomes [22]. On the other hand, personalized medicine utilizes genetic information to pinpoint specific targets for therapy, such as mutations in cancerous cells. This approach allows for treatments that directly target the disease mechanisms. Many common and complex diseases are influenced by the collective effects of multiple genes [18,48]. Personalized medicine has the potential to transform healthcare from reactive to proactive (see Table 1 below). In personalized medicine, diagnostic testing is extensively employed to identify the most effective medications for a patient based on their genetic profile or other molecular and cellular investigations. Key tools in personalized medicine include molecular diagnostics, imaging techniques, and advanced analytics [49,50].

Personalized medicine enables doctors to more accurately predict which medications will be effective for specific conditions, thereby benefiting patients significantly. This approach represents a new clinical paradigm and a fundamental shift in medicine within the healthcare system. By enhancing drug selection and tailoring treatments, personalized medicine minimizes adverse effects, improves patient compliance, shifts the medical focus from reactive response to proactive prevention, increases cost efficiency, and strengthens patient trust in approved treatments [51].

Actress Angelina Jolie’s reaction to inheriting the *BRCA* gene highlights the significant impact of advanced genomic information on understanding disease risks and preventive options. Her public disclosure has not only increased awareness and promoted proactive healthcare but also sparked crucial conversations about the ethical, psychological, and societal implications of genetic testing. Following Jolie’s disclosure, often referred to as the “Angelina Jolie Effect”, there was a noticeable rise in the number of women seeking genetic testing for *BRCA* gene mutations. Jolie’s announcement drew widespread attention to the *BRCA1* and *BRCA2* gene mutations, which greatly elevate the risk of breast and ovarian cancer. This increased awareness contributed to a broader public understanding of genetic risk factors for cancer susceptibility [52,53].

So many case studies today illustrate how personalized medicine is transforming healthcare by utilizing genetic and molecular information to tailor treatments. By aligning therapies with individual patients, healthcare providers can enhance outcomes, minimize side effects, and improve the overall effectiveness of treatment plans. As personalized medicine continues to evolve, it promises further advancements in patient care and increased efficiency within health systems [54]. For instance, a patient with cardiovascular disease was found to carry a variant in the *CYP2C19* gene, which affects the metabolism of clopidogrel, a common antiplatelet medication. Instead of the standard clopidogrel, the healthcare provider prescribed a different antiplatelet agent that was more effective for the patient’s genetic profile. This adjustment reduced the risk of adverse events and improved the patient’s overall treatment adherence [55]. This case highlights the potential of pharmacogenomics to optimize drug therapies, reduce adverse effects, and improve patient compliance, leading to better health outcomes and more efficient healthcare delivery.

By emphasizing prevention and early intervention mechanisms, personalized medicine has the potential to decrease the occurrence of chronic diseases and alleviate associated healthcare burdens [56]. This transition from reactive to proactive healthcare holds promise for enhancing public health outcomes and lowering long-term healthcare expenses. In addition to genetic information, personalized medicine takes into account family history and lifestyle factors, offering a comprehensive evaluation of an individual’s risk for chronic diseases. This stratified risk assessment allows for more precise and targeted prevention strategies. Individuals identified as high-risk can benefit from preventive medications and therapies tailored to their specific needs. For instance, individuals at high risk for heart disease may receive prescriptions for statins or other medications aimed at reducing cholesterol levels and blood pressure [51,57].

Personalized medicine utilizes biomarkers to detect early signs of chronic diseases before they become clinically apparent. Early detection allows for timely interventions that can prevent disease complications [58]. Advanced technologies enable continuous monitoring of biomarkers and other health indicators. This ongoing surveillance aids in the early detection of changes indicating the onset of a chronic disease, facilitating prompt medical interventions. Early intervention through personalized medicine decreases the necessity for costly treatments, hospitalizations, and long-term care typically associated with advanced stages of chronic diseases. By averting or postponing the onset of chronic conditions, personalized medicine improves individuals’ quality of life. Patients experience reduced symptoms, maintain better physical function, and enjoy longer, healthier lives [59,60]. However, to fully harness the potential of personalized medicine and ensure equitable access to its benefits, societal, ethical, and logistical challenges must be effectively addressed [61].

## 4. Future Synergies Between Multi-Omics Data and Precision Medicine

Modern advancements in personalized medicine heavily rely on technologies that confirm a patient’s fundamental biology, such as DNA, RNA, or protein analysis, which are essential for accurate disease diagnosis [62]. As technology continues to advance, the synergies between DNA analysis and precision medicine are expected to expand, unlocking new potentials for disease prevention, treatment, and management. For instance, personalized techniques like genome sequencing can reveal DNA mutations that impact diseases ranging from cystic fibrosis to cancer [63].

**Figure 1:**
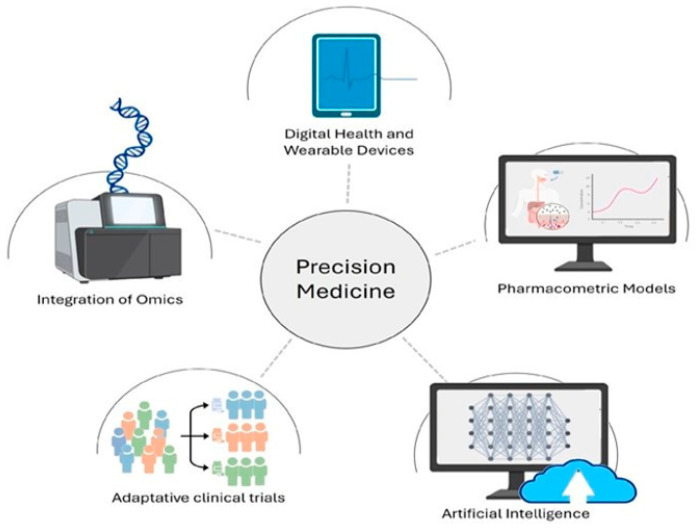
Key elements of in silico approaches in precision medicine. Created with Biorender.com. Available online: http://biorender.com/ (accessed on 19 August 2024).

Omics sciences study the collective characterization of biological molecules, providing insights into an organism’s structure, function, and dynamics. In precision medicine, multi-omics integrates data from genomics, transcriptomics, proteomics, metabolomics, and more to uncover how individual molecular differences influence disease development, progression, and treatment responses. This holistic approach helps unravel complex biological processes and disease mechanisms, supporting personalized healthcare (Figure 1 shows integrative approaches to data from a multi-omics level) by tailoring treatments to an individual’s unique molecular makeup [64].

Currently, several international networks and projects are focused on applying multi-omics approaches to precision medicine, such as The Cancer Genome Atlas (TCGA), The Precision Medicine Initiative (PMI) Cohort Program, Pan-Cancer Analysis of Whole Genomes (PCAWG), and the Human Proteome Project (HPP) [65]. For example, the European Joint Programme on Rare Diseases (EJP RD), a Europe-wide initiative, aims to accelerate the diagnosis and treatment of rare diseases by integrating multi-omics data to better understand disease mechanisms and enhance therapeutic precision [65].

Early detection through genetic screening can prompt preventive measures, lifestyle adjustments, and ongoing monitoring to identify diseases in their early stages. Another technique, called RNA-seq, can unveil the RNA molecules implicated in specific disease conditions. Unlike DNA, RNA levels can fluctuate in response to environmental conditions. Therefore, sequencing RNA can offer a more comprehensive understanding of a person’s health status and conditions. Recent investigations have connected genetic differences between individuals to RNA expression [66], translation [67], and protein levels [68,69].

In a 2018 study on pancreatic ductal adenocarcinoma, researchers combined genomics, transcriptomics, and proteomics data to identify distinct molecular subtypes that exhibit varied responses to treatment, enhancing the classification of patients for personalized therapy [70]. Similarly, a 2020 study applied a multi-omics approach to cerebrospinal fluid samples from patients with Alzheimer’s, integrating proteomics and metabolomics data to uncover new biomarkers associated with the disease, deepening the understanding of its molecular mechanisms [71]. These examples illustrate how multi-omics approaches have transformed the understanding of complex biological systems and diseases, improving diagnostic and therapeutic strategies across various fields.

### 4.1. The Role of Genomics in Personalized Medicine

Genomics empowers personalized medicine by integrating genetic information into routine clinical decision-making with the goal of optimizing patient outcomes and enhancing healthcare efficiency. It plays a crucial role in personalized medicine by offering insights into an individual’s genetic makeup. Patient-specific medical approaches, based on innovative technologies and scientific discoveries, have transformed medical practices and consequently the healthcare system. Recent advancements in biotechnology have led to the creation of advanced diagnostic devices capable of detecting genetic mutations, protein concentrations, and other biomarkers with exceptional precision. Currently, advancements in next-generation sequencing (NGS) technologies have substantially reduced the cost and time needed for whole-genome and exome sequencing. This makes it easier to conduct detailed analysis of unique genetic profiles, identifying mutations and genetic variations associated with diseases [72,73].

Genomics plays a crucial role in identifying biomarkers for diseases such as cancer, enabling early diagnosis, monitoring, and personalized treatments. Cancer treatment, in particular, benefits significantly from genomic analysis and precision medicine. Genomic analysis helps pinpoint specific genetic mutations that drive cancer development. By analyzing these mutations unique to a patient’s cancer, targeted treatments can be tailored to eliminate cancer cells more effectively while minimizing damage to healthy tissue. For example, mutations in the BRCA1 and *BRCA2* genes serve as important biomarkers for breast and ovarian cancers [74,75].

For example, gene therapy and editing technologies such as CRISPR enable precise modifications to DNA. These techniques involve introducing, removing, or altering genetic material within a person’s cells to treat or prevent disease. They hold the potential to correct genetic defects at their origin, potentially offering cures for genetic disorders that were previously untreatable [76]. In general, the convergence of genomics, biotechnology, and data analytics is driving personalized medicine forward, presenting transformative potential for healthcare systems. By facilitating more precise, predictive, and preventive strategies, personalized medicine is expected to enhance patient outcomes and elevate the overall state of public health [77].

### 4.2. The Role of Pharmacogenomics in Personalized Medicine

Another significant aspect central to personalized medicine is pharmacogenomic testing, which analyzes an individual’s genetic profile to predict how they will respond to medications. This personalized approach helps tailor treatments to maximize effectiveness and minimize adverse effects based on genetic variations that affect drug metabolism and response. By integrating pharmacogenomic testing into personalized medicine practices, healthcare providers can optimize medication selection and dosing procedures. This integration enhances medication quality control and therapeutic efficacy by tailoring treatments based on individual genetic profiles, thereby improving patient outcomes and reducing adverse effects [3,78].

Depending on our genome, individuals respond differently to various drugs. For example, some people may absorb medications too quickly, requiring higher doses to achieve therapeutic effects, while others metabolize them slowly, leading to an increased risk of side effects. A study involving 7000 participants, published in February, demonstrated that tailored doses of certain drugs based on pharmacogenetic testing significantly reduced the occurrence of side effects [3,79].

Genome-informed prescribing is arguably one of the earliest areas to showcase the transformative impact of personalized medicine on a broad scale [80]. The capability to offer real-time recommendations relies on developing machine-learning algorithms that predict which patients are likely to benefit from specific medications based on their genomic information. The essence of personalizing medications and dosages lies in genotyping patients before the need for such information arises [81].

Using genetic information to inform medication selection and dosing enables healthcare providers to mitigate the risk of adverse drug reactions and enhance treatment outcomes. Pharmacogenomic testing also aids in identifying patients who may not respond well to standard medications, enabling the implementation of more precise and effective treatment approaches [82]. For example, rapid and precise diagnosis of seriously ill infants suspected of having a genetic disease can be achieved through the use of rapid whole-genome sequencing combined with NLP-enabled automated phenotyping [83]. The integration of pharmacogenomics into routine clinical care still faces significant barriers. These obstacles range from fundamental pharmacogenomics research to the practical implementation in clinical settings.

Studying previously overlooked rare genetic variants and validating their functional and clinical implications through the development of pre-clinical models and in silico tools is essential for advancing pharmacogenomic knowledge. Concurrently, international coordinated efforts aimed at overcoming current barriers to pharmacogenomic implementation are expected to yield new tools and insights for its clinical application, paving the way for broader adoption across healthcare systems [84,85].

Medicine has long aimed to achieve this goal, and advancements in genomics hold promise for facilitating this endeavor. Identifying individual susceptibility to certain diseases and responses to therapy is a crucial step toward implementing optimal strategies for disease prevention and care [86]. By integrating various aspects of DNA study, personalized medicine strives to deliver more precise, predictive, and preventive healthcare, thereby enhancing patient outcomes overall.

### 4.3. The Role of Transcriptomics in Personalized Medicine

Transcriptomics, which examines the entire set of RNA transcripts generated by the genome under specific conditions, is pivotal in personalized medicine. This field primarily aims to understand gene expression patterns and their impact on individual health and disease conditions. It involves analyzing the transcriptome, encompassing all RNA classes like mRNA, rRNA, tRNA, and non-coding RNAs. Methods such as RNA sequencing (RNA-seq) are commonly used to measure gene expression levels and detect phenomena such as alternative splicing, RNA editing, and the functionality of non-coding RNAs [87,88].

Currently, transcriptomic profiles can uncover specific gene expression patterns associated with various diseases, aiding in precise diagnosis. Transcriptomics is being investigated as a complementary method to genomic testing for precision-based treatments in patients with cancer. This approach helps identify molecular signatures that inform treatment decisions, potentially improving therapeutic outcomes by tailoring treatments based on gene expression profiles [89].

Transcriptomics is instrumental in distinguishing between different cancer types and subtypes. Changes in gene expression can serve as prognostic markers, aiding in predicting disease progression and patient outcomes. Additionally, transcriptomics can identify biomarkers that predict how patients will respond to specific treatment strategies. This facilitates the selection of the most effective treatment while minimizing adverse effects. By revealing the dynamics of gene expression linked to disease conditions, researchers can identify novel therapeutic targets personalized to an individual’s transcriptomic profile [89,90].

Furthermore, transcriptomic data can reveal the activation or suppression of specific biological pathways in disease conditions. This insight can guide the development of targeted treatment strategies aimed at addressing underlying molecular dysfunctions [91]. In addition to this, exploring the interactions between different genes and their regulatory elements aids in understanding complex diseases and identifying points for intervention. Transcriptomics, when combined with other multi-omics data such as genomics, proteomics, and metabolomics, enables a comprehensive analysis of biological processes underlying health and disease. This integrative approach enhances the accuracy and effectiveness of personalized medicine treatment strategies. Overall, transcriptomics greatly enhances personalized medicine by offering detailed insights into gene expression patterns and their implications for health and disease conditions [92].

### 4.4. The Role of Proteomics in Personalized Medicine

Proteomics, the comprehensive study of proteins, including their structures and functions, plays a crucial role in advancing personalized medicine by providing insights into individual health and disease variations. Proteins are essential components in biological processes, and understanding their dynamics can shed light on disease mechanisms and treatment responses. Proteomics involves analyzing the proteome, which encompasses all proteins expressed by a genome, cell, tissue, or organism at a specific time. Techniques such as mass spectrometry and protein microarrays are commonly used in proteomics to detect, quantify, and analyze protein modifications and interactions. This detailed analysis helps uncover biomarkers, understand disease pathways, and develop targeted therapies tailored to individual patients [93,94].

Proteomic analysis plays a crucial role in disease diagnosis by identifying protein biomarkers associated with specific disease conditions, enabling early and precise detection. For example, certain cancers and cardiovascular diseases exhibit unique protein expression profiles that proteomics can distinguish between, thereby differentiating disease subtypes and informing treatment selection based on disease nature [95]. Moreover, proteomics analyzes protein expression and modifications to predict patient responses to specific drugs, facilitating the selection of effective therapies while minimizing adverse effects. This personalized approach enhances treatment efficacy and improves patient outcomes in clinical practice. Furthermore, studying protein–protein interactions can yield insights into complex biological processes and how they are disrupted in disease states. Proteomics plays a critical role in identifying proteins involved in drug action, metabolism, and resistance. This knowledge contributes to the development of new drugs personalized to individual molecular profiles, thereby improving treatment effectiveness and patient outcomes [96].

In general, proteomics significantly enhances personalized medicine by offering detailed insights into protein expression, function, and interactions. This enables precise disease diagnosis, customization of treatments, and deeper understanding of disease mechanisms, ultimately leading to more effective and individualized healthcare solutions. The integration of proteomics with other omics technologies holds promise for advancing personalized medicine further, improving patient outcomes, and transforming medical practice.

### 4.5. The Role of Metabolomics in Personalized Medicine

Metabolomics, the in-depth study of metabolites within a biological system, holds a crucial role in personalized medicine. It encompasses the detection and quantification of small molecules (metabolites) in cells, tissues, or bodily fluids. These metabolites serve as the end products of cellular processes, and their levels offer insights into the physiological state of an organism [5]. By analyzing the full spectrum of metabolites within a biological sample, metabolomics can identify biomarkers that signify the presence or progression of diseases like cancer, cardiovascular diseases, and metabolic disorders. This approach enables early detection and personalized treatment strategies based on metabolic profiles [97]. Metabolomics enables early and precise diagnosis, aiding in distinguishing between different subtypes of a disease. This capability is crucial for selecting appropriate treatments tailored to individual patients.

Additionally, through the analysis of metabolic profiles, clinicians can monitor patient responses to medications and make necessary dosage adjustments. Metabolomics also can also identify altered metabolic pathways in disease conditions, potentially revealing new therapeutic targets. Personalized nutritional plans can be tailored based on individual metabolic profiles, optimizing health outcomes and managing conditions such as diabetes and obesity. Moreover, metabolomics data can predict which patients may benefit from specific drugs and who might experience adverse effects, allowing for customized pharmaceutical options [98,99].

Metabolomics facilitates continuous monitoring of health status by detecting deviations from normal metabolic states, providing early warnings of potential health issues. Importantly, metabolomics is often integrated with other omics methods, such as genomics, transcriptomics, and proteomics, to offer a comprehensive understanding of biological processes and enhance personalized medicine strategies. This multi-omics approach can elucidate complex interactions among genes, transcripts, proteins, and metabolites, thereby enabling more precise and individualized medical interventions [99,100].

### 4.6. The Role of Epigenomics in Personalized Medicine

Epigenomics is the study of the complete set of epigenetic modifications on the genetic material of a cell, known as the epigenome. It plays a crucial role in understanding gene regulation and cellular identity within a multi-omics framework. By combining epigenomic data with other omics layers, researchers can achieve a more comprehensive view of biological systems, elucidate disease mechanisms, and identify potential therapeutic strategies [101]. This field is crucial in personalized medicine because epigenetic changes can be influenced by factors such as environment, lifestyle, and chemical exposures. These changes are mediated by mechanisms like DNA methylation, histone modification, and non-coding RNA molecules, which can affect how genes are activated or silenced without changing the DNA sequence itself. Epigenetic variations can have significant impacts on health and the development of diseases [102].

Epigenetics provides insights into additional regulatory mechanisms and environmental exposures beyond an individual’s unique genome. It reveals personal history through biological markers, allowing for personalized approaches to drug regimens, disease susceptibility, and treatment outcomes based on epigenetic profiles. This information enhances our understanding of individual health risks and responses to therapies, contributing to more tailored and effective healthcare strategies in personalized medicine [103].

For instance, drugs targeting epigenetic modifications, such as DNA methyltransferase inhibitors and histone deacetylase inhibitors, are under development and used to treat various cancers and other diseases. These therapies can be tailored to an individual’s epigenetic profile, enhancing their effectiveness. Epigenetics also provides insights into how genes are regulated in different disease states, thereby identifying molecular mechanisms underlying diseases and potential new therapeutic targets. This knowledge contributes to advancing personalized medicine by offering targeted treatments based on epigenetic profiles and improving outcomes for patients with complex diseases [104,105].

Furthermore, studying epigenetics can uncover how environmental factors and lifestyle choices such as diet, smoking, alcohol consumption, and stress affect gene expression and influence the development of diseases [106]. A thorough analysis of an individual’s epigenetic profile can inform personalized recommendations for lifestyle and diet to enhance health and mitigate disease risks. Epigenetics plays a crucial role in advancing personalized medicine by offering comprehensive insights into gene regulation and its implications for health and disease management. Epigenetics is facilitating more accurate diagnosis, personalized treatment strategies, and a better understanding of disease mechanisms. By combining with other multi-omics technologies, it holds the potential for significant progress in personalized medicine, enhancing patient outcomes and pushing medical practice to new heights [107]. The below Figure 1 clearly shows how different tools are integrated together to achieve the aims of precision medicine in our health care.

### 4.7. The Role of Phenomics in Personalized Medicine

Phenomics is the extensive study of phenotypes, which are the observable physical and biochemical traits of organisms. This field analyzes how these traits are shaped by a combination of genetic, environmental, and developmental factors. By providing a thorough understanding of the interplay between individual phenotypes and their genetic and environmental influences, phenomics plays a vital role in the advancement of personalized medicine [108].

**Figure 2:**
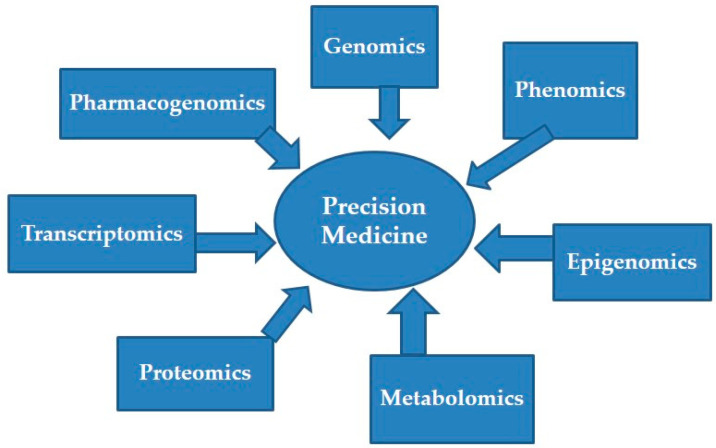
Multi-omics data for the advancement of precision medicine.

By integrating genetic, phenotypic, environmental and many other omics data (see Figure 2), phenomics enhances risk assessment models for various diseases, facilitating earlier interventions. This approach enables healthcare to transition from a one-size-fits-all model to more individualized strategies that optimize patient care and improve outcomes. Ultimately, this comprehensive understanding has the potential to revolutionize healthcare, making it more proactive, predictive, and tailored to the needs of each patient [108].

## 5. Applications of Personalized Medicine

Personalized medicine involves tailoring treatment strategies to an individual’s genome and specific needs, potentially enhancing outcomes and minimizing side effects by taking into account genetic, environmental, and lifestyle factors. This approach could lead to earlier diagnosis, intervention, and the development of more effective drugs and targeted therapies. As a result, personalized medicine represents a paradigm shift from traditional treatment approaches, promising significant advancements across various healthcare domains [109].

### 5.1. Oncology

Oncology is particularly focused on personalized medicine due to the intrinsic variability of cancer. Cancer is a complex disease marked by mutations in key genes, leading to changes in molecular pathways. Genetic tests in oncology have implications for both healthy individuals and those already affected by cancer. For healthy individuals, screening tests can predict the risk of developing certain disorders. Meanwhile, for patients with cancer, molecular profiling provides insights into the disease’s onset, progression, and response to treatment, enabling tailored medication strategies for improved outcomes [38].

Hereditary cancers constitute approximately 10–15% of all cancers, yet extensive studies of the human genome have facilitated the identification of cancer risk factors and the discovery of prognostic and predictive biomarkers. Additionally, research has uncovered genetic variants in drug-metabolizing genes, which aid in understanding drug toxicity and predicting responses to therapy [110].

Currently, the connection between genomic variations and various cancers is well established. For instance, mutations in genes such as *BRCA1* and *BRCA2*, as well as *PARP* genes, are associated with breast cancer tumors. The *ERBB2* receptor is linked to breast adenocarcinoma, the *BCR/ABL* fusion gene to chronic myelogenous leukemia, and *BRAF* (*V600E*) mutations to melanoma, colorectal cancer, and thyroid cancer. These associations highlight the importance of genetic factors in understanding and treating these types of cancer [111].

Moreover, genetic tests offer valuable information for optimizing therapy through targeted treatments. For example, Herceptin is used in patients with female breast cancer with *HER-2* expression, Imatinib targets the *BCR/ABL* fusion gene in chronic myeloid leukemia, Vemurafenib targets *BRAF* mutations in melanoma, and Gleevec inhibits tyrosine kinase activity in chronic myeloid leukemias. These targeted therapies illustrate the benefits of genetic testing in tailoring treatment approaches based on specific genetic characteristics of tumors [10,112]. This approach enhances treatment strategies by personalizing them, resulting in improved treatment outcomes compared to conventional methods that prescribe the same drug for patients with varying genetic profiles.

Despite numerous studies already conducted, the ongoing discovery of new genetic variants continues. The ability to identify actionable or druggable alterations within each tumor using advanced technologies is paving the way for personalized medicine in oncology [113].

### 5.2. Diagnosis and Intervention

The advent of personalized medicine brings forth numerous opportunities, notably advancing and refining disease diagnosis. Molecular-based diagnostic tests are increasingly accessible, enabling the establishment of more effective treatment strategies. By evaluating each patient individually, healthcare providers can achieve more precise diagnoses and tailor treatment plans accordingly. Genotyping involves molecular tests that identify an individual’s DNA sequence. By comparing this genetic code to a reference genome, such as that from the Human Genome Project, healthcare professionals can identify existing genetic variations that may play a role in disease susceptibility or progression [38,109].

This information can be used to diagnose and treat individuals effectively based on their genetic background. Understanding one’s genetic makeup is essential to predict their response to specific treatments, potentially altering the type of treatment received to improve effectiveness. Pharmacogenomics exemplifies this approach, using an individual’s genome to tailor drug prescriptions more precisely and knowledgeably [114].

Diagnostic tests play a crucial role in guiding therapy within the framework of personalized medicine, often forming part of a theranostic platform. These tests encompass various medical imaging techniques, including MRI contrast agents, fluorescent markers, and nuclear imaging agents such as PET radiotracers or SPECT agents [16], as well as in vitro lab tests with nucleic acid (DNA or RNA) sequencing of certain samples [17], along with deep learning bioinformatics tools and AI algorithms that weigh the results of tests for several different biomarkers [115].

To assess the effectiveness and safety of a drug, companion diagnostic tests tailored to specific patient groups or sub-groups can now be conducted. This technology is designed to improve therapeutic options for individuals based on assay results developed either during or after the drug’s release to market [19]. Companion diagnostics has integrated pharmacogenomics information into drug prescription labels, helping prescribers make informed and effective treatment decisions for their patients [116].

### 5.3. Pharmacogenomics

Pharmacogenomics examines how an individual’s genetic composition influences their response to medications. It merges pharmacology, the study of drugs, with genomics, the exploration of genes and their roles. As research progresses and technology becomes more available, the incorporation of pharmacogenomics into everyday medical care is expected to grow, fostering a more personalized approach to healthcare. Pharmacogenomics aims to create personalized medicine strategies by customizing drug treatments according to individuals’ genetic profiles. This approach enhances treatment efficacy and mitigates the risk of adverse drug reactions [3,117].

Understanding how genetic variations influence individuals’ responses to medications is crucial due to the unique genetic profiles, lifestyles, and environmental factors of each person. Variations in DNA sequences can impact how drugs are metabolized, influencing their efficacy and safety differently across individuals. This knowledge aids in prescribing medications that are not only more effective but also have fewer side effects tailored to each individual’s genetic makeup [118]. On the other hand, establishing the correct drug dosage based on genetic makeup, as seen with medications like warfarin and other anticoagulants, significantly impacts treatment outcomes, both positively and negatively. Therefore, it is crucial to administer the appropriate dose tailored to each patient’s genetic profile. For example, cytochrome P450 enzymes, which are pivotal in drug metabolism, exhibit genetic variations among individuals that can result in varied drug responses [119].

### 5.4. Cardiovascular Diseases

Certain genetic markers can predict susceptibility to cardiotoxicity caused by drugs used in treating various conditions, including chemotherapy agents. Recognizing these markers enables the selection of safer alternative treatments or the implementation of more rigorous monitoring protocols. Today, genetic markers can also assess risks, identifying individuals at higher risk for conditions such as heart disease. This facilitates early intervention and preventive measures, potentially averting more severe complications later on [119].

Currently, personalizing treatment plans for conditions like hypertension, hyperlipidemia, and other cardiovascular diseases based on genetic predispositions is being implemented. For example, Warfarin, an anticoagulant used to prevent and treat thromboembolic events, poses challenges in dosing due to its narrow therapeutic range and considerable variability among patients. Genetic variations in genes such as *VKORC1* and *CYP2C9* play a significant role in influencing both the sensitivity to warfarin and its metabolism in individuals [120]. Pharmacogenetic testing can assist in establishing the most suitable starting dose, thereby reducing the risk of bleeding or thrombosis and achieving stable anticoagulation more quickly. This personalized approach tailors medical treatment to each patient’s individual characteristics, including their genetic makeup, resulting in potentially more effective and safer therapeutic outcomes [121].

### 5.5. Rare Genetic Disorders

The incorporation of personalized medicine into the management of patients with rare genetic disorders not only enhances outcomes but also deepens understanding of these complex conditions, facilitating the development of innovative treatments and potential cures. Whole-genome sequencing is a valuable tool in elucidating rare genetic diseases, enabling diagnoses that may be elusive using traditional techniques [122]. Genetic screening plays a crucial role for families with a history of rare genetic disorders, identifying carriers and guiding reproductive decisions. Prenatal testing detects genetic abnormalities early, enabling timely interventions or informed choices. Advances in gene therapy have allowed for the correction of underlying genetic defects, such as in spinal muscular atrophy (SMA), a rare neuromuscular disorder. For example, the FDA-approved gene therapy drug Zolgensma delivers a functional copy of the *SMN1* gene to patients [123].

### 5.6. Neurological Disorders

In the realm of neurological disorders, personalized medicine holds the potential to greatly improve diagnosis, treatment, and management. By taking into account the distinct genetic, environmental, and lifestyle factors of each patient, personalized medicine can tailor approaches more effectively. Identifying genetic mutations and variations linked to neurological disorders aids in early diagnosis and the development of personalized treatment strategies [124]. For instance, mutations in the *HTT* gene are associated with Huntington’s disease, while mutations in the *APP*, *PSEN1*, and *PSEN2* genes are linked to early-onset Alzheimer’s disease. Today, researchers can use genetic information to predict the risk of developing Alzheimer’s disease and customize preventive strategies accordingly [125].

Integrating neuroimaging data with genetic profiles and clinical information enables the development of predictive models for forecasting disease onset, progression, and treatment outcomes in neurological disorders such as Alzheimer’s and Parkinson’s diseases. Imaging tools like MRI and PET scans offer detailed views of brain structure and function. Personalized medicine leverages these images in conjunction with genetic data to gain deeper insights into disease mechanisms in individual patients, enhancing precision in diagnosis and treatment strategies [126].

The implementation of personalized medicine in neurological disorders offers significant potential for enhancing patient treatment outcomes. Through the integration of genetic, molecular, and clinical data, personalized medicine enables more accurate diagnosis, precise treatment targeting, and effective disease management. Ongoing progress in genomics, neuroimaging, and computational biology will continue to refine and advance the ability to personalize medical care for individuals with neurological conditions [126,127].

### 5.7. Infectious Diseases

As technologies and methodologies progress, the incorporation of personalized medicine into the management of infectious diseases is expected to become more widespread, resulting in enhanced patient treatment outcomes and public health benefits [128]. Advanced genomic sequencing techniques such as next-generation sequencing (NGS) can rapidly pinpoint the exact pathogen responsible for an infection. This swift identification enables healthcare providers to select targeted antimicrobial therapies more accurately, thereby shortening the time to appropriate treatment. In-depth genetic analysis of pathogens, including distinguishing between bacterial or viral strains, plays a crucial role in understanding the source of infection, transmission patterns, and potential mechanisms of drug resistance [129].

Genomic profiling can identify resistance genes within pathogens, aiding in the selection of antibiotics that are likely to be effective against specific strains. For example, identifying the *mecA* gene in *Staphylococcus aureus* indicates methicillin resistance (MRSA), prompting healthcare providers to choose alternative antibiotics [130]. Understanding the resistance profile of a pathogen enables healthcare providers to avoid prescribing ineffective antibiotics and instead select treatments to which the pathogen is susceptible. This approach improves treatment outcomes, minimizes unnecessary medication costs, and helps curb the spread of resistant strains [131].

For instance, whole-genome sequencing of *Mycobacterium tuberculosis* can pinpoint drug-resistant strains, informing the selection of effective drug combinations and helping to prevent the spread of resistant TB [132]. Another example is HIV treatment, where customizing antiretroviral therapy based on both the genetic makeup of the virus and the patient’s own genetic factors has proven beneficial. In the case of malaria, genetic testing for G6PD deficiency, which can lead to severe reactions to certain antimalarial drugs like primaquine, assists in selecting safer treatment options [133].

### 5.8. Diabetes Management

Personalized medicine holds great promise for managing diabetes by customizing treatment strategies to each patient’s unique characteristics. Genetic testing can identify individuals at high risk of developing type 1 or type 2 diabetes. Specific genetic variants, such as those in the *HLA* region for type 1 diabetes and *TCF7L2* for type 2 diabetes, facilitate early risk assessment and inform preventive strategies [134].

Genetic variations can indeed affect how patients respond to diabetes medications. For example, variations in the *CYP2C9* gene can influence the metabolism of sulfonylureas, potentially affecting their effectiveness and increasing the risk of hypoglycemia. This highlights the importance of personalized medicine in tailoring diabetes treatment to individual genetic profiles [135]. Customizing drug choices and dosages according to these genetic insights can significantly enhance treatment outcomes. Genetic markers, such as variants in the *SLC22A1* gene, can predict the efficacy of metformin, which is a primary treatment for type 2 diabetes. This personalized approach helps optimize diabetes management by selecting treatments that are likely to be most effective for each individual [136].

Personalized dosing strategies play a crucial role in optimizing blood glucose control and minimizing side effects in diabetes management. Tailored insulin regimens can be developed using continuous glucose monitoring (CGM) data and individual patient factors. This approach allows for precise adjustments in insulin dosage and timing to achieve improved glycemic control while reducing the risk of hypoglycemia. Biomarkers such as HbA1c, fasting glucose levels, and C-peptide levels provide valuable guidance for therapy adjustments, helping to prevent complications associated with diabetes [137,138].

Personalized dietary recommendations can indeed be tailored based on genetic and metabolic profiles. For example, individuals with specific genetic predispositions may derive greater benefits from low-carbohydrate diets or diets customized to their insulin sensitivity. Similarly, personalized exercise plans can be formulated by taking into account individual metabolic responses, fitness levels, and genetic factors. This personalized approach not only enhances glucose control and weight management but also reduces cardiovascular risk [139].

### 5.9. Drug Development

Personalized medicine is transforming drug development by making it more precise, efficient, and aligned with the individual needs of patients, ultimately leading to more effective treatments and better healthcare outcomes. By understanding the genetic underpinnings of diseases, researchers can pinpoint new drug targets. This allows them to concentrate on specific genetic mutations or pathways that drive disease progression [140]. By identifying biomarkers through genomic and proteomic analyses, which indicate the presence or severity of a disease, researchers can develop targeted therapies more effectively.

Personalized medicine allows for the selection of patient subgroups that are more likely to respond to a drug, which improves the efficiency and success rate of clinical trials. Trials can be designed to adapt based on interim results, such as adjusting dosages or targeting different subgroups, resulting in quicker and more cost-effective studies. Tailoring treatments to individual genetic profiles also helps minimize the likelihood of adverse reactions, thereby enhancing the safety and predictability of the drug development process [141].

Drugs developed using a personalized approach are often more effective and have higher efficacy rates because they are designed to target specific genetic or molecular profiles [84]. Personalized medicine that focuses on specific subgroups can also reduce the overall cost of drug development by shrinking the size and duration of clinical trials. Additionally, personalized medicine can uncover new genetic contexts where existing drugs may be effective through repurposing trials, potentially extending the indications and patents of established medications. Regulatory agencies are increasingly acknowledging the value of personalized medicine and adjusting guidelines to facilitate the development and approval of targeted therapies [140].

## 6. Main Challenges of Personalized Medicine

Personalized medicine holds promise for significant advancements in healthcare but faces several challenges that impede its widespread adoption and effectiveness. Table 2 below clearly shows the drawback of both personalized and conventional medicine on the ground. These hurdles encompass technical, ethical, regulatory, and economic domains. Successful implementation of personalized medicine necessitates collaboration across diverse fields, including genomics, bioinformatics, clinical medicine, and data science. However, integrating these disciplines can be challenging due to disparities in terminology, methodology, and cultural practices [142]. Addressing the challenges of personalized medicine requires coordinated efforts from researchers, clinicians, policymakers, and other stakeholders within the healthcare ecosystem. Collaboration among these groups is essential to overcoming technical, ethical, regulatory, and economic obstacles and to successfully implementing personalized medicine for the benefit of patients [143].

### 6.1. Data Privacy and Security

Personalized medicine depends significantly on genetic data and other sensitive health information. Protecting data privacy and ensuring security are vital in this field because of the sensitivity of the data used. Genetic details are extremely private and can disclose much about a person’s health, disease risks, and even their lineage. If this information is accessed without authorization, it can result in severe breaches of privacy. It is essential to safeguard the privacy and confidentiality of genetic data to prevent the potential misuse of individuals’ information. The extensive personal genetic information needed for personalized medicine heightens concerns about data privacy and security. Mishandling this data can result in discrimination by employers and insurance companies or even lead to social stigmatization. Preventing breaches of this sensitive information is a significant challenge [144].

The practice of personalized medicine frequently involves data sharing among various entities, such as research institutions, healthcare providers, and third-party service providers. Consequently, it is crucial to establish clear and secure data sharing agreements. However, the varying standards for data security across different systems and platforms make it challenging to maintain consistent protection. Securely storing large volumes of genetic data requires advanced technology and substantial resources [145]. The security protocols of cloud storage solutions must also be thoroughly evaluated. Determining the appropriate retention period for genetic data and ensuring its secure disposal when it is no longer needed are crucial for minimizing risks and preventing information leaks to unauthorized parties. Addressing these challenges necessitates a multi-faceted approach, which includes implementing robust encryption methods, conducting regular security audits, enforcing stringent access controls, establishing comprehensive privacy policies, and providing ongoing education and training for all stakeholders involved in personalized medicine [146].

Minimizing ethical considerations and data privacy issues in personalized medicine necessitates a comprehensive approach that balances patient autonomy with the need for innovation. To achieve this, several strategies can be employed: first, when sharing genomic data internationally, it is essential to comply with the most stringent data privacy standards, such as the General Data Protection Regulation (GDPR), and to anonymize surnames before data sharing across jurisdictions. Adhering to regulations like GDPR and the Health Insurance Portability and Accountability Act (HIPAA) is crucial. Additionally, there should be a transparent consent process, ensuring that patients are fully informed about how their data will be used, stored, and shared. Furthermore, all research involving personal data must undergo an ethical review to confirm adherence to established standards. By implementing these strategies, personalized medicine can progress while maintaining ethical boundaries and safeguarding patient data privacy [147,148].

### 6.2. Ethical and Social Issues

The development of personalized medicine, which customizes medical treatment to each patient’s unique characteristics, holds great promise for enhancing public healthcare outcomes. However, it also brings up several ethical and social concerns that must be carefully addressed. Greater public education about personalized medicine is needed to help people understand its benefits and limitations, as misunderstandings could lead to unrealistic expectations or unnecessary fears. Additionally, different cultures have varying views on genetics and medical interventions [149].

Personalized medicine must be culturally sensitive and consider diverse values and beliefs. While it has the potential to significantly improve healthcare outcomes, addressing the associated ethical and social issues is crucial for its responsible development and implementation. This requires multidisciplinary strategies involving ethicists, policymakers, healthcare providers, and the public to create frameworks that ensure ethical standards, equitable access, and societal trust in personalized medicine. Ethical issues include potential discrimination based on genetic information, ensuring informed consent for genetic testing, and the psychological impact of knowing one’s genetic risks [150].

Various ethical issues are associated with personalized medicine. Firstly, access to advanced medical technologies often varies significantly between high-income and low-income countries. Additionally, most genomic research has historically focused on populations of European descent, leading to significant underrepresentation of racial and ethnic minorities. This disparity in representation can affect the algorithms used for drug selection and dosing, potentially resulting in ineffective treatment and poorer outcomes for patients with different genetic backgrounds and lifestyles. Therefore, ensuring that personalized medicine is developed and implemented in an ethical, equitable, and socially responsible manner is crucial to realizing its full potential [149,151].

### 6.3. Regulatory Hurdles

There are several regulatory hurdles in the development and implementation of personalized medicine. These challenges stem from the need to ensure the safety, efficacy, and ethical use of personalized treatments while balancing innovation with public protection. Adapting regulatory frameworks to the complexities of personalized medicine is a multifaceted task that requires innovative approaches and continuous adaptation [152]. By developing more flexible, collaborative, and ethically sound regulatory pathways, we can create an environment that supports the safe and equitable advancement of personalized medicine. This will ultimately help to maximize the benefits of personalized treatments while addressing the ethical and social concerns associated with this transformative field. Currently, regulatory frameworks are not fully adapted to the complexities of personalized medicine. Approving new treatments and diagnostics based on genetic information requires rigorous validation and standardization [153].

Personalized medicine frequently involves highly specialized treatments, such as gene therapies, which pose challenges for traditional regulatory frameworks. Traditional clinical trials are typically designed for broader populations, whereas personalized medicine focuses on specific subgroups. This necessitates new trial designs capable of effectively evaluating the safety and efficacy of treatments for smaller, more targeted populations. The complexity of these therapies makes establishing standardized guidelines challenging. Regulatory agencies could enhance flexibility by creating adaptive pathways that accommodate the unique aspects of personalized medicine [19,152].

To address these challenges, regulatory bodies could consider implementing conditional approvals or accelerated review processes for innovative treatments in personalized medicine. These approaches can help expedite access to therapies while ensuring safety and efficacy. Additionally, fostering collaboration between various fields involved in personalized medicine is crucial. This could involve establishing interdisciplinary panels or advisory boards to provide expertise and guidance for regulatory decisions [51]. Furthermore, efforts to harmonize regulations internationally should be intensified. This can be achieved through international agreements, collaborative research initiatives, and the development of global standards. By working together across borders, regulatory agencies can facilitate smoother and more efficient pathways for the approval and adoption of personalized medicine treatments worldwide [154].

### 6.4. Economic Barriers

Economic obstacles pose a substantial challenge to the advancement and adoption of personalized medicine within healthcare systems. These barriers can hinder access to healthcare, affect its delivery, and challenge its long-term sustainability. They also impede the development, implementation, and accessibility of personalized treatments, which rely on expensive technologies like high-throughput sequencing machines and bioinformatics tools for therapeutic planning [155]. Genetic testing and personalized treatments often come with prohibitive costs. Furthermore, insurance coverage for these tests and treatments varies significantly, impacting accessibility for many individuals. Personalized treatments, such as gene therapies and targeted drugs, typically necessitate extensive research and lengthy clinical trials, which ultimately contribute to the overall development expenditures [156].

Determining prices for personalized treatments remains difficult due to their high development expenses and the imperative to ensure affordability. Overcoming the economic barriers to personalized medicine demands a comprehensive strategy involving enhanced funding, policy adjustments, innovative pricing strategies, and a commitment to equity. By addressing these challenges, personalized medicine can become more accessible and sustainable, thereby enhancing healthcare outcomes for diverse populations globally [157].

### 6.5. Integration into Clinical Practice

Incorporating personalized medicine into routine clinical practice requires a comprehensive overhaul of healthcare infrastructure. This transformation involves significant investments in technology, changes in clinical protocols, and extensive training for healthcare professionals to ensure effective implementation. Key aspects of this integration include updating clinical guidelines and providing healthcare professionals with the necessary skills and knowledge [158].

In the future, medical and nursing schools should integrate genomics and personalized medicine into their curricula. This includes providing training in genetic counseling, interpreting genomic tests, and understanding the ethical, legal, and social implications of personalized medicine within healthcare systems. Ongoing education should emphasize interdisciplinary collaboration, as personalized medicine frequently requires cooperation among geneticists, bioinformaticians, pharmacologists, and clinicians [159]. This involves establishing standardized protocols for genetic testing and the application of personalized treatments to ensure consistency and reliability across various healthcare settings. It is also crucial to implement robust data management systems capable of handling the large volumes of genetic data generated. This includes ensuring secure storage, efficient retrieval, and advanced analysis capabilities [160].

### 6.6. Interdisciplinary Collaboration

Personalized medicine requires collaboration across diverse disciplines, such as genomics, bioinformatics, various medical specialties (like oncology, cardiology), pharmacology, and clinical medicine. This collaborative approach facilitates the sharing of knowledge and expertise in applying personalized medicine approaches across different domains. Coordinating these efforts can be complex and challenging, but it can lead to innovative advancements in healthcare system development. For example, genomic scientists conduct in-depth analyses of patients’ genetic data to identify mutations and genetic variants that affect disease susceptibility, drug responses, and treatment outcomes [161].

Effective collaboration necessitates genomics experts sharing detailed genetic data with bioinformaticians and clinicians to integrate genetic insights into patient care systems seamlessly. Pharmacologists play a crucial role in researching and developing drugs that target specific genetic profiles, determining optimal dosage regimens, enhancing treatment efficacy, and reducing side effects. Therefore, collaborating with pharmacologists to understand how genetic variations impact drug metabolism and effectiveness is vital for advancing healthcare systems tailored to each individual [162].

Each discipline brings specialized knowledge, insights, and tools that, when integrated, improve the precision and effectiveness of medical treatments customized for individual patients. Bioinformaticians develop tools and algorithms to manage and analyze extensive genomic datasets, identifying significant patterns and correlations. They construct models that predict how genetic variations influence disease progression and response to treatment. By harnessing the distinct expertise of each discipline, healthcare can evolve to be more precise, effective, and patient-centered. This interdisciplinary approach not only improves treatment outcomes but also stimulates innovation, optimizes resource allocation, refines diagnostic pathways, and promotes ethical practices in personalized medicine [163,164].

### 6.7. Scientific Challenges

There are numerous scientific challenges that must be tackled for the effective development and integration of personalized medicine into real healthcare systems. These challenges encompass scientific research, technology, data analysis, and clinical application. One major hurdle comprehends the intricate relationships among genes, environment, and lifestyle in disease progression and treatment efficacy. Progress in analytical tools, extensive data collection, and interdisciplinary cooperation is crucial for unraveling these relationships [165].

Addressing these challenges is crucial for refining personalized medicine to offer more effective and customized healthcare solutions, ultimately enhancing patient outcomes and public health. It is important to note that various genetic mutations can lead to the same disease, and conversely, the same mutation can result in different diseases or phenotypes. Additionally, many diseases are influenced by multiple genes, each contributing in small ways, which complicates predicting disease risk and response to treatment [166].

Integrating data from genomics, transcriptomics, proteomics, metabolomics, and other omics technologies is intricate and demands sophisticated computational tools. Managing and analyzing large-scale datasets from diverse sources necessitates advanced data storage, processing, and analytical capabilities. Understanding the interactions between genetic predispositions and environmental factors (like diet, pollutants, and lifestyle) is crucial yet challenging due to the complexity and variability of these interactions [167].

The human microbiome, a highly complex ecosystem, plays a significant role in health and disease conditions, yet its interactions with host genetics and environmental factors are still not fully understood. Moreover, identifying and validating biomarkers that accurately predict disease risk, progression, and treatment response with high specificity and sensitivity poses a substantial challenge. Although next-generation sequencing has become more accessible, ensuring accuracy, reproducibility, and cost-effectiveness remains an ongoing hurdle [168].

Addressing these challenges requires a multidisciplinary approach involving collaboration among geneticists, clinicians, bioinformaticians, ethicists, and policymakers. By overcoming these obstacles, personalized medicine can fully realize its potential in improving healthcare outcomes and enhancing quality of life [169]. This advancement promises to improve patient outcomes and elevate healthcare delivery to new heights.

## 7. Future Perspectives for the Personalized Medicine

The future of personalized medicine holds the potential to transform healthcare by moving away from a standardized approach to one that customizes treatments based on individual genetic, environmental, and lifestyle factors. The decreasing cost and faster pace of whole-genome sequencing, driven by high-throughput technologies, will enhance accessibility and enable comprehensive genetic profiling of individuals. This vast genomic dataset will facilitate more accurate identification of genetic predispositions to diverse diseases [22].

As technology and research progress, personalized medicine will increasingly integrate into everyday clinical practice, potentially enhancing healthcare outcomes and patient quality of life. Achieving this future entails ongoing collaboration among genomic scientists, researchers, clinicians, policymakers, and patients to navigate challenges and maximize the benefits of personalized healthcare [22,170].

### 7.1. Advancements in Genomic Medicine

Genomic medicine, which harnesses the genome’s capabilities to diagnose, treat, develop therapies for currently untreatable genetic conditions, and prevent diseases, has made significant strides in recent years. These advancements hold great promise for revolutionizing healthcare by enabling more precise diagnoses, assessing risks, personalizing treatments, and introducing innovative therapeutic strategies. As research progresses, the integration of genomic information into routine clinical practice is expected to become commonplace, ultimately improving health outcomes for patients globally [171].

Today, whole-genome sequencing (WGS) is becoming more commonplace as the cost continues to decrease and the sequencing time shortens, making it increasingly viable for clinical use. This advancement allows for more accurate identification of genetic mutations associated with diseases [172]. For example, polygenic risk scores (PRS) are utilized to evaluate the risk of complex diseases by analyzing the collective impact of numerous genetic variants. This capability can aid in early detection and prevention strategies for conditions such as heart disease, diabetes, and psychiatric disorders. Moreover, CRISPR and other gene-editing technologies have the potential not only to identify genetic defects but also to correct them, offering avenues to treat genetic disorders at their fundamental cause [173].

### 7.2. Artificial Intelligence and Machine Learning

Artificial intelligence is a paradigm shift toward precision medicine [174]. Today, AI and machine learning can process large volumes of genomic and clinical data, uncovering patterns and correlations that might elude human observation. Machine learning algorithms are employed to analyze genomic sequences and draw insights from extensive patient and healthcare institution data collected continuously. This capability improves diagnostic precision and enables personalized treatment plans tailored to individual patients [175].

At present, AI is employed to forecast disease risks and outcomes using genetic and environmental data, facilitating earlier and more precise interventions. Personalized risk assessments help inform lifestyle adjustments and medical interventions aimed at preventing disease onset. Furthermore, AI integrates data from genomics, transcriptomics, proteomics, and metabolomics to offer a comprehensive understanding of an individual’s health status. AI techniques are applied in precision cardiovascular medicine to analyze genotypes and phenotypes in established diseases. This application aims to enhance patient care quality, promote cost-effectiveness, and lower rates of readmission and mortality [175].

The advancement of artificial intelligence (AI) and machine learning (ML) is transforming cancer treatment through personalized therapeutic choices. AI innovations examine genetic and molecular profiles of tumors to pinpoint the most effective targeted therapies. Meanwhile, ML models forecast which patients are probable to benefit from immunotherapy, thereby enhancing treatment outcomes [175,176].

### 7.3. Biomarker Discovery

Proteomics, the study of proteins on a large scale, and metabolomics, the study of small molecules or metabolites on a large scale, are emerging fields with promising potential to investigate cellular, fluid, or tissue changes that provide deeper insights into disease processes. Progress in these fields is expected to uncover new biomarkers for diseases, enabling early diagnosis and more effective monitoring of treatment outcomes. Biomarkers encompass genomic, proteomic, metabolic, or imaging-based indicators that reflect biological processes or responses to therapies [71]. For instance, identifying single nucleotide polymorphisms (SNPs) associated with disease susceptibility and drug response aids in disease prediction and prevention. Copy number variations (CNVs) also play a role by influencing gene expression and contributing to disease development, serving as valuable diagnostic markers. Additionally, epigenetic markers such as DNA methylation and histone modification patterns are used to diagnose and predict the progression of diseases like cancer. These biomarkers collectively enhance our ability to understand and manage diseases more effectively [177].

Developing personalized panels of biomarkers customized to individual genetic and environmental backgrounds has the potential to significantly enhance the precision of diagnosis and treatment effectiveness. Progress in biomarker discovery, fueled by high-throughput technologies, artificial intelligence, and collaborative research, is facilitating more precise and personalized medical interventions [178].

### 7.4. Collaborative Research and Data Sharing

The advent of personalized medicine represents a new opportunity for scientists from diverse backgrounds to collaborate and advance global healthcare. It has the potential to greatly enhance collaborative research and promote data sharing, fostering innovation and improving healthcare outcomes. International collaboration and data sharing are crucial for understanding genetic diversity across populations and developing treatment strategies that can be universally applied. This collective effort is essential for leveraging the full potential of personalized medicine in shaping the future of healthcare worldwide [154,179].

Collaborative research projects often bring together experts from various domains to develop comprehensive personalized treatment strategies. This pooling of expertise from different fields can yield novel insights and breakthroughs that might not be achievable in isolation. By promoting collaborative research and sharing of data, personalized medicine is poised to drive innovations in healthcare. This approach holds the promise of delivering more effective and customized treatments to patients worldwide [180].

### 7.5. Tailored Therapeutics

The goal of personalized medicine is to enhance treatment outcomes and minimize adverse effects that are significant to both clinicians and patients. It represents a paradigm shift in healthcare, moving beyond the traditional one-size-fits-all approach to a more individualized and precise method of disease treatment. Patients are treated according to their individual circumstances [3]. One mechanism for developing tailored treatments involves identifying genetic markers that predict how a patient will respond to a specific drug. This approach ensures that only those likely to benefit receive the medication, thereby improving treatment outcomes. Another strategy is developing therapies that target specific molecular pathways involved in a patient’s disease, which can increase the likelihood of successful treatment. Additionally, understanding how genetic variations affect drug metabolism helps in avoiding medications that may cause harmful side effects in certain individuals, thereby preventing serious illness related to medications [181].

Additionally, personalized medicine facilitates the identification of individuals at high risk for specific diseases, enabling preventive measures and early interventions. Making personalized medicine more accessible and affordable will ensure that more patients can benefit from tailored therapeutics. The future of personalized medicine holds promise for transforming healthcare by enhancing precision, predictiveness, effectiveness, and customization to individual needs. However, realizing this potential necessitates ongoing innovation, adequate research funding, collaboration across disciplines, and careful consideration of ethical, legal, and social implications [182,183].

## 8. Conclusions and Recommendation

Precision medicine allows healthcare providers to better understand factors like environment, lifestyle, and genetics that influence a patient’s health. The goal is to match treatments precisely to a patient’s unique profile. Advances in genomics over the past two decades have deepened our understanding of how genetic variations affect health. Personalized medicine, which tailors treatments based on a patient’s genetics, could reduce side effects and improve outcomes. In the future, integrating complete genomic profiles into healthcare records could help clinicians predict and treat diseases more effectively. The pharmaceutical industry is expected to shift towards specialized, personalized treatments, with genomics playing a crucial role. Developed countries are already working to collect global genomic data, with COVID-19 accelerating the development of personalized medicine. The aim is to shape future healthcare systems by developing personalized medicines. The following includes some specific recommendations for future research or actions to advance the field:

- Focus on building comprehensive genomic databases that include diverse populations from different ethnic backgrounds.
- Explore effective algorithms and platforms for real-time integration of genomic data into clinical workflows, investigating how clinicians can use this data for personalized diagnosis, treatment planning, and preventive care.
- Encourage pharmaceutical companies to prioritize pharmacogenomics in drug development to create treatments that are tailored to specific genetic profiles, minimizing side effects and improving efficacy.
- Explore AI-driven models that integrate genomic, environmental, and lifestyle data to develop more accurate disease prediction models.
- Conduct studies to assess the ethical challenges surrounding genomic data usage, focusing on data ownership, consent, and privacy.

## Figures and Tables

**Table 1:** Comparison between personalized medicine and conventional medicine.

Features of Personalized Medicine	Features of Conventional Medicine
Utilizes a patient’s genetic profile	Relies on standardized treatments
Targeted therapies	Designed for broad groups of people
Predictive and preventive healthcare	Focus on disease diagnosis and treatment
Biomarker-based diagnostics	Treatments and interventions are based on scientific research, clinical trials, and rigorous testing
Customized treatment plans	Pharmaceuticals (drugs) and surgical interventions are central
Incorporates large-scale data analysis, such as “big data” from electronic health records	Relies heavily on advanced diagnostic tools and technologies, such as X-rays and MRI scans
A patient-centric approach	Medical practice is guided by clinical guidelines
Precise dosage of medications	Drug dosages and treatment protocols are generally standardized
Besides genetics, personalized medicine integrates lifestyle factors	Focuses on managing symptoms

**Table 2:** Comparisons of the drawbacks of personalized and conventional medicine.

Drawbacks of Personalized Medicine	Drawbacks of Conventional Medicine
High cost for diagnosis, drug development and insurance coverage	Costs can rise due to unnecessary diagnostic tests or treatments
Genetic data privacy issue	Less focus on data privacy
Limited availability	More likely available
Complexity in implementation	One-size-fits-all approach
Regulatory and legal challenges	Strict regulation
Relies on advanced technologies and specialized knowledge	Symptom-focused
Lack of expertise but advancing quickly	Healthcare inequities and slow to adopt new technologies or practices
Equity in access to treatment	Antibiotic resistance and surgical risks
Predictive testing	Reactive, not preventive

## Abbreviations

**AI**	**Artificial Intelligence**
CML	Chronic myelogenous leukemia
CNVs	Copy number variation
DNA	Deoxyribonucleic acid
EHRs	Electronic health records
MRI	Magnetic resonance imaging
NGS	Next-generation sequencing
PET	Positron emission tomography
RNA	Ribonucleic acid
SNPs	Single nucleotide polymorphism
Glossary	
Epigenomics	The study of chemical changes and modifications to DNA and associated proteins that affect gene expression without altering the underlying DNA sequence.
Genomics	The study of an organism’s entire genetic material (genome), including the structure, function, evolution, and mapping of genes.
Metabolomics	The study of the complete set of small molecules, or metabolites, within a biological sample.
Omics	A collective term for fields of study in biology that focus on large-scale data analysis of molecules, such as genes (genomics), proteins (proteomics), or metabolites (metabolomics), to understand biological systems and processes comprehensively.
Personalized medicine	A medical approach that tailors treatment and healthcare decisions to an individual’s unique genetic makeup, lifestyle, and environment, with the goal of providing more effective and targeted therapies.
Pharmacogenomics	The study of how an individual’s genetic makeup affects their response to drugs.
Precision medicine	A medical model that customizes healthcare by considering individual variability in genes, environment, and lifestyle to develop more accurate and effective treatments and preventive strategies for patients.
Proteomics	The large-scale study of the entire set of proteins produced by an organism or cell.
Transcriptomics	The study of the complete set of RNA transcripts produced by the genome under specific conditions or in a particular cell type.
